# Hysteresis in Pressure-Driven DNA Denaturation

**DOI:** 10.1371/journal.pone.0033789

**Published:** 2012-04-09

**Authors:** Enrique Hernández-Lemus, Luz Adriana Nicasio-Collazo, Ramón Castañeda-Priego

**Affiliations:** 1 Computational Genomics Department, National Institute of Genomic Medicine, México, D.F., México; 2 Center for Complexity Sciences, National Autonomous University of México, México, D.F., México; 3 Division of Sciences and Engineering, University of Guanajuato, León, Guanajuato, México; Universidad Veracruzana, Mexico

## Abstract

In the past, a great deal of attention has been drawn to thermal driven denaturation processes. In recent years, however, the discovery of stress-induced denaturation, observed at the one-molecule level, has revealed new insights into the complex phenomena involved in the thermo-mechanics of DNA function. Understanding the effect of local pressure variations in DNA stability is thus an appealing topic. Such processes as cellular stress, dehydration, and changes in the ionic strength of the medium could explain local pressure changes that will affect the molecular mechanics of DNA and hence its stability. In this work, a theory that accounts for hysteresis in pressure-driven DNA denaturation is proposed. We here combine an irreversible thermodynamic approach with an equation of state based on the Poisson-Boltzmann cell model. The latter one provides a good description of the osmotic pressure over a wide range of DNA concentrations. The resulting theoretical framework predicts, in general, the process of denaturation and, in particular, hysteresis curves for a DNA sequence in terms of system parameters such as salt concentration, density of DNA molecules and temperature in addition to structural and configurational states of DNA. Furthermore, this formalism can be naturally extended to more complex situations, for example, in cases where the host medium is made up of asymmetric salts or in the description of the (helical-like) charge distribution along the DNA molecule. Moreover, since this study incorporates the effect of pressure through a thermodynamic analysis, much of what is known from temperature-driven experiments will shed light on the pressure-induced melting issue.

## Introduction

The molecule of Deoxyribonucleic acid (DNA) is a very complex one, both from the physicochemical point of view, as well as its obvious biological function. In recent times many of its dynamic and structural features have re-attracted the attention of the scientific community. Clearly, the number of sophisticated experiments (spectroscopical, biochemical, mechanical tests, among others), many of them at the one-molecule level, has increased [1]–[3] whereas deep theoretical studies ranging from continuous mechanics to quantum chemistry and statistical physics abound [4]–[7]. The variety of available experimental and theoretical findings is thus almost endless see, e.g., [8]–[10] and references therein. Nevertheless, there is a lack of a conceptual framework to categorize and analyze this enormous bunch of information. A thermodynamic theory seems to be the ideal candidate for doing such a task. Since many of the interesting features of the behavior of DNA are dynamic in nature and due to the fact that many of them occur in a mesoscopic scale, a non-equilibrium thermodynamic treatment is therefore appropriate. Of course, such a general theory does not seem to be at hand at the moment. Nonetheless, in this work we will try to establish some grounds of it, specifically with respect to the problem of DNA melting, since it is one of the most important features which is closely related with the biological function of genetic transcription [11], [12].

Nowadays, it is well understood that the static structure of complex biological molecules is not sufficient to explain their functions. A well-established fact is that such biomolecules, specifically proteins and nucleic acid heteropolymers reveal, apart from the usual molecular vibrations, purely stochastic transitions between a multitude of conformational substates [13]. This fact is particularly true for the DNA molecule which undergoes large conformational changes during transcription or replication. Several physiological reasons are behind such a large number of conformational states. On one hand, such a large number of conformational states is needed for the selective catalysis mechanisms involved in the highly accurate mechanisms of genomic expression. On the other hand, at a fundamental physical level, it has been possible to show experimentally that a single chromosome is made up of one DNA molecule that is some 4 to 10 cm long (see [4] and references 4 and 5 therein). So that the entire genome contained on a human cell is about 2 m long. For this reason the genome is highly compacted in a variety of complex arrays within each cell [11], [12]. The ternary structure of DNA has been proved to be helicoidal (the double helix); the length of the molecule is much larger than its diameter, so it is common to describe it as a one dimensional deformable string [5], [14], [15] or, sometimes, like a highly charged rod [16]. Such an oversimplification is useful only when one is not interested in phenomena related to its internal structure [17]. Additionally, the fundamental biological features of the DNA function are replication and transcription; both phenomena are so complex that a fundamental (physical) description of them is unavailable at the moment. However, DNA melting or denaturation -which is a related phenomenon- can be more easily studied from a molecular point of view within a thermodynamical conceptual framework. Having seen that the *reading* of the genetic code requires a great number of complex conformational changes one is aware that DNA possesses an *ever changing* structure that determines its own function which is essential for the existence of life as we know it [18]. Nowadays, it is important to study the dynamics of such biomolecules.

The aim of this work is, therefore, to present a thermodynamic theory that accounts for the phenomenon of hysteresis during the denaturation process of DNA molecules. This theory, which combines an irreversible thermodynamics approach with an equilibrium mean-field approximation for the osmotic pressure of the DNA suspension, is able to predict hysteresis curves for a DNA sequence in terms of its configurational states as well as system parameters like salt concentration, density and temperature. From a pure non-equilibrium thermodynamics point of view, the use of a microscopic formalism to predict equilibrium macroscopic properties is not a common task, i.e., equations of state or constitutive equations are typically taken from experiments. Hence, the formalism here discussed represents our proposal of understanding the denaturation phenomenon of charged biopolymers through the coupling of two different approaches. Furthermore, the equation of state (EOS) here employed is obtained by means of a microscopic route that naturally allows us to manipulate the physical parameters and, then, permits us to account for its effects on the denaturation process of DNA molecules. The use of the so-called PB-cell mean-field approximation to describe the osmotic pressure of a DNA suspension is justified explicitly in the work of Hansen et al. [16], where a cell model description of the ionic atmosphere near a cylindrical polyelectrolyte predicts osmotic properties that are in surprisingly good harmony with all available experimental findings over a wide range of DNA concentrations. However, a more accurate EOS of a suspension of DNA molecules will to take into account some characteristics of DNA, such as the helical-like charge distribution, in order to incorporate important electrostatic effects. Recently, it has been shown that the mean-field theory breaks down at short separation between two doubled stranded DNA molecules [17]. However, the present work studies a low DNA concentration regime, similar to the one present in cells under *in vivo* conditions (recall that the approximate content of DNA in a single cell is in the 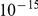-mol [femto-mol] order of magnitude [19]); hence even this relatively simple degree of approximation has been able to capture the essence of the complex behavior in pressure-driven DNA denaturation.

The paper is organized as follows. First, a short introduction to the study of the DNA molecule as a complex mechanic and thermodynamic entity is given. We briefly review some of the main theoretical approaches to the problem. Some experimental techniques that have been applied to describe the dynamics of DNA denaturation are described. Then, the paper focuses on the description of the main physical and chemical mechanisms responsible for the complex thermodynamical behavior of the DNA molecule at its melting transition. In the next section, we are faced with the non-equilibrium behavior of DNA within the context of an irreversible thermodynamic framework. After that, the Poisson-Boltzmann mean-field approximation, within the cell model, for the calculation of the osmotic pressure of a DNA suspension (here modeled as a suspension of charged cylindrical objects, see, e.g., Ref. [16]) is summarized. As a result we outline a possible mechanism behind the hysteretic behavior of the DNA melting transition based on the presence of irreversible coupling between the chemical potential (as given by the hydrogen bond interactions in the *backbone*) and the local stress distribution. Finally, we discuss our results, as well as some of their implications, limitations and future premises.

## Analysis

### Denaturation Dynamics in DNA

**Figure 1:**
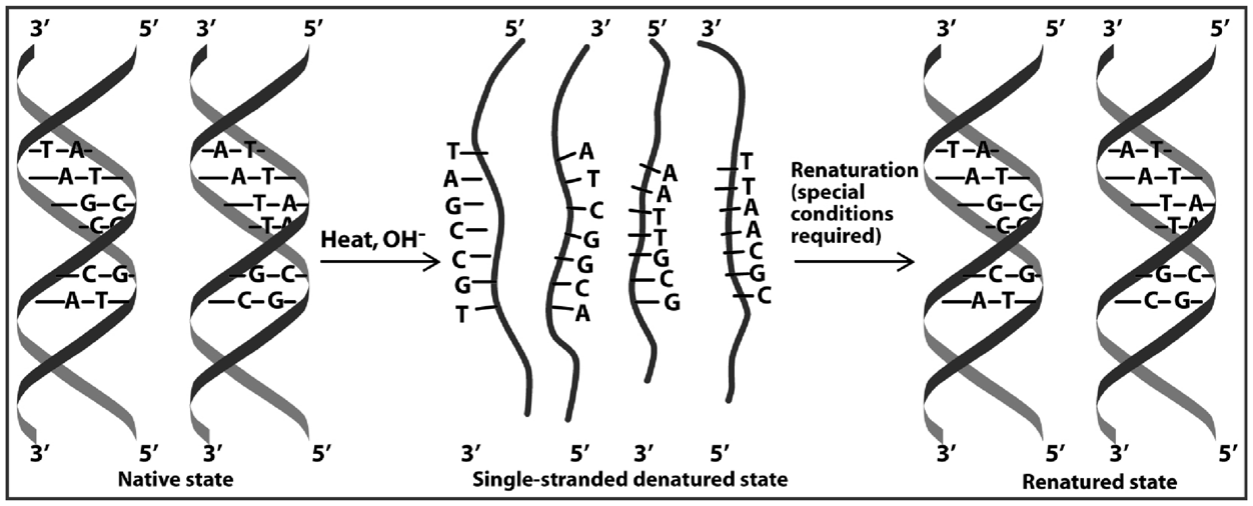
DNA denaturation or melting. Native DNA on its double-helix configuration (left) undergoes a phase change due to the action of chemical or thermal agents to a single-stranded *denaturated* state (center). Under special conditions the double-helical configuration can be recovered (right).

One of the first steps in the study of the molecular biophysical behavior of the DNA molecule is to understand the mechanisms for DNA denaturation or melting. DNA melting is the separation of the two strands of the double helix by heating, or changing other parameters such as salt concentration or hydrostatic pressure [20], [21] (see Figure 1). It has been easy to monitor the dynamical process of melting experimentally, since the breaking of the hydrogen bonds between base pairs is accompanied by a very large increase of the absorbance of ultraviolet light at nearly 260 nm [20], [21]. These experiments show that denaturation occurs in multiple steps and that the process is highly sensitive to the given sequence order [22]. In fact, DNA denaturation is a collective effect determined by at least hundreds of base pairs, this fact has made very difficult to understand many of the experimental data and relate them to a theoretical approach. Recently, theoretical findings seem to point out to the presence of large amplitude fluctuations that may play an important role in the transition [4]. In order to take this large fluctuations into account there one has to consider the non-linear mechanisms present in the process such as the entropic (configurational) and torsional effects in the DNA chain. Recently, the role of stochasticity in the ternary structure of DNA has been studied in the framework of the Stochastic Matrix Method (SMM), an iterative stochastic model, and related to the nonlinearities in the process of DNA melting [22]. One of the advantages of this method is that is parameter-free. Although it works out well in predicting the static properties of the denaturation process, one is still in the need for a model to explain its dynamic properties. In addition, a mesoscopic model for heterogeneous DNA denaturation is proposed within the framework of the time-dependent path integral formalism [7].

It is well known that local DNA denaturation is an obligatory step for the initiation of the transcriptional process at the molecular level, also involved in other genetic functions such as DNA replication. Due to this fact, it is expected that the loci and dynamics of denaturation should be strictly controlled. Of the possible controlling mechanisms one that stands up for its own importance is the role of mechanic stress at the double helix. For example, sites predicted to be susceptible to stress-induced duplex destabilization (a preliminary step for denaturalization) have been found to be closely associated with some specific transcriptional regulatory regions [23]. According to sequence-dependent molecular stress studies, even though mechanisms of DNA function often involve complex interactions with the chemical environment, the intrinsic susceptibility to the so-called stress induced base unpairing at specific sites is essential for biological activity [15], [23], [24]. In Figure 1 we could observe DNA in a denatured state (i.e. singe-stranded DNA), this conformation is the one that is necessary for biological information-flow since, either *information-copying* (replication) or *information-encoding* (transcription) is carried-out in (and from) ss-DNA.

Nowadays, several experimental groups report that double stranded DNA (dsDNA) undergoes a stress induced phase transition to a so called overstretched form (S-DNA), when exposed to a stretching force of about 65 piconewtons [4], [6], [15]. This *non-equilibrium phase transition* is remarkably sharp on its presence, always between 60 and 70 piconewtons depending on salt concentration (the so-called ionic strength). It has been mentioned [6] that this S-DNA is in fact strand separated, giving thus rise to a theory of tension driven strand separation (stress induced melting or tension melting). Further molecular mechanics studies have revealed that although S-DNA has some of its hydrogen bonds broken, its behavior is not that of single stranded DNA (ss-DNA). This seems to point out to the possibility that S-DNA could be in an intermediate stage between ds-DNA and ss-DNA. i.e. S-DNA could be in a denaturalized state [14], [15]. There exists an enormous quantity of experimental, theoretical and computational data on this class of mechanic phenomena on DNA [1]–[3], [5], [11], [12], unfortunately, some of these findings are even in mutual contradiction. It is our belief that if we embed this enormous corpus of information within a general theoretical framework, the source of these seeming contradictions will be unveiled.

There is of course, additionally, an important biological motivation in understanding thermo-mechanical effects on the structure and function of DNA. It has been stated that the binding of proteins and drugs to double-stranded DNA is weaker under high hydrostatic pressure and also that the activity and selectivity of enzymes as RNA polymerase depend upon the pressure [25]. In what follows we will formulate some basic playground to begin the systematic study of these phenomena in the light of irreversible thermodynamics.

### Thermodynamic Formalism

As it is usual in Irreversible Thermodynamics (IT), we shall start our discussion by assuming that a generalized entropy-like function 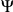 exists, which may be written in the form [26]:
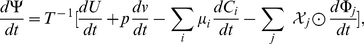
(1)


or in terms of differential forms
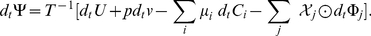
(2)


Equation (2) resembles the proposal made by Chen and Eu [27] that
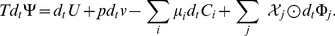
(3)


We observe that equations (2) and (3) are just the formal extension of the Gibbs equation of equilibrium thermodynamics for the case of a multi-component non-equilibrium system. The quantities appearing therein are the standard ones, i.e.,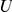 is the internal energy, 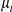 is the local chemical potential, 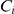 the local concentration for species 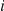, 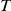 is the local temperature, 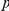 and 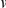 the pressure and volume, respectively. 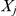 and 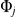 are thermodynamical fluxes and forces. The latter ones take into account the aforementioned non-local effects. 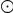 is the most general scalar product. As it is known, classical theories for memory effects have successfully connected transport processes with fluctuations and stochasticity. A general description is given in reference [26], where the authors show precisely the equivalence of theories for transport with memory and generalized entropies such as the ones used in the present paper and in most of the extended irreversible thermodynamics formulations [28].

Prigogine’s theorem states from stability conditions on equation (3) (namely that a nonequilibrium steady state is characterized by a constant value of 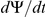) that if one of the terms in equation (3), say the stress contribution, changes it must be related to a change in another term, say the concentration gradient, in order to ensure the continuity and stationarity of 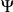. This will lead the system to an steady state or will maintain the stability of the system in this steady state. In the case of hysteresis in DNA denaturation, there is a strong experimental evidence supporting this claim (see, e.g., [3]–[6]). Hence, the main features of the physical phenomena of hysteresis were identified, i.e., the existence of internal dissipation processes. These features could be taken into account in a non-equilibrium thermodynamics analysis. Given a kinetic model for the denaturation dynamics and experimental data (a phenomenological law) on the rate of change of the stress (in the present case the hydrostatic pressure), we will be able to write down a closed expression for the system representing the effect of hysteresis.

### Poisson-Boltzmann Formalism

Biological structures such as biopolymers, membranes and cellular components normally contain a large number of charged groups so that they may be conceptualized as *macroions*. Additionally, there is a large variety of smaller ions (microions) in the medium, such as Na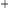, Cl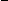, K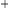, etc., strongly bound to macroions forming the so-called double layer. Coulombic interactions thus play an important role in the formation of structures and in transport processes which are fundamental for the function of biological entities [29]. The large charge asymmetry between macro-ions and micro-ions gives rise to the condensation effect of the latter ones on the macro-ion surface. This strong accumulation of small ions around a big ion leads to interesting phenomena, such as the macro-ion charge renormalization or the screening Coulomb interaction [30], [31]. This non-linear effect not only affects the interaction potential between macroions, it is the responsible for the system thermodynamics in systems with weak screening [32]. Moreover, the evaluation of the system pressure in highly charged systems becomes a very demanding, almost impossible, task since the average force acting on each particle must be computed to evaluate the equilibrium equation of state through the virial route [31]. One typically overcomes this situation by using mean-field approximations based on the well-known Poisson-Boltzmann formalism [32].

Once we know the local charge distribution we are able to calculate the *electrostatic potential* 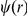. This potential is the cornerstone of any electrostatic theory, included the Poisson-Boltzmann mean field theory. In order to obtain 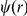 we can use Poisson-Boltzmann equation as follows [30],
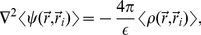
(4)where 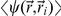 and 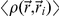 are the average electrostatic potential and local charge-density, respectively. The latter can be expressed as,
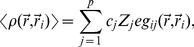
(5)



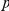
 being the number of charged species, 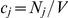 the bulk concentration of 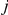 ions, 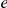 the fundamental charge, 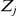 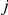-species valence and 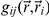 is the radial distribution function for 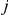-ions around a central 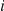-ion located at 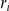. In thermodynamic equilibrium under the so-called Debye-Hückel approximation (i.e. microions uniformly distributed as a cloud around the macroion), 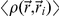 could be written as follows [33]

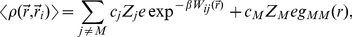
(6)where 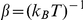 is the inverse thermal energy, 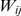 is the 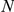-body potential of mean force, 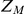 is macroion’s valence and 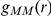 is the radial distribution function among macroions. By using (6), we could re-write PB equation as
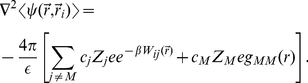
(7)


Using a heuristic approximation, which has been extensively used in the symmetric electrolyte case, the potential of mean force can be expressed in terms of the electrostatic potential as
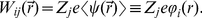
(8)


Equation (8) is a closure under the symmetric-electrolyte approximation to rewrite PB equation as follows
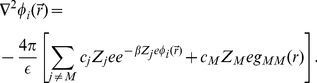
(9)


Expression (9) is the full non-linear Poisson-Boltzmann equation. To obtain a solution of equation (9) is a hard task and can be only achieved numerically. One can then gain further information of its solution by studying a few simple limiting cases. For instance, assuming a single macroion, i.e., infinite dilution limit, and linearizing the r.h.s. of equation (9), i.e.,
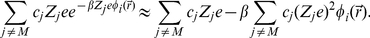
(10)one obtains,
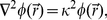
(11)since by electro-neutrality the first term of the r.h.s. of equation (10) is zero and,
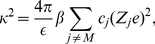
(12)is called the Debye-Hückel screening parameter. Unfortunately, although one can obtain an analytical solution of equation (11) in a few geometries, its solution is only valid for an *infinitely diluted system*. Therefore, to solve the PB equation (9) one needs to apply further approximations. The simplest one is to consider a particular form, a priori unknown, of the radial distribution function between macroions. This kind of approximation has been successfully applied for describing the thermodynamics properties of highly charged colloids in suspension and is usually called the PB-cell model, see e.g., [30], [34] and references therein.

#### PB-cell model

**Figure 2:**
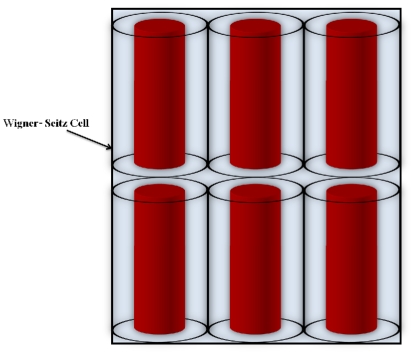
Wigner-Seitz cell model (6 cells shown) for DNA molecules under PB cell model. Every cylinder has a radius 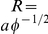 with 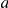 the radius of a DNA molecule, i.e. 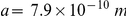, and 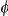 as stated is the volume fraction.

PB-cell model [34]–[36] rests on the observation that repulsively interacting biomolecules arrange their positions such that each biomolecule has a region around it which is void from other biomolecules and which looks rather similar for different biomolecules. In other words, the Wigner-Seitz cells around arbitrarily selected biomolecules are comparable in shape and volume. One now assumes that the total charge within each cell is exactly zero, that all cells have the same shape, and that one may approximate this shape such that it matches the symmetry of the biomolecule, i.e., cylindrical cells around cylindrical biomolecules (for a geometrical depiction see Figure 2). The cell radius 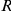 is chosen in consistency with the particle number density, 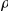, and the PB equation within the cell is solved with appropriate boundary conditions at the cell edge and the biomolecule surface. Thus, through the finiteness of the cell plus the boundary conditions, the presence of all those biomolecules not inside the cell are taken into account. Poisson-Boltzmann approximation depends on the structure (or geometry) of the biomolecule and the parameters of the medium such as pH, temperature and ionic strength [29].

PB equation can be written as
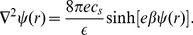
(13)It is nevertheless more useful to write down PB equation in terms of the dimension-less electrostatic potential 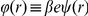, to give the expression,
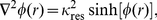
(14)where 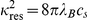 is the inverse screening length, a function of the ionic strength (or salt concentration) 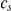, and 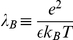 the Bjerrum length [30], [34].

DNA is modeled as a cylindrical object with a linear distribution, 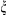, of charge along the symmetry axis; Figure 2 shows a schematic representation of our DNA model. Such a model has shown its validity [29] in the regime where the length (or persistence length) of the DNA chain is larger than the screening length, 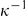. For example, in 1 mM of NaCl, DNA has a persistence length of about 1000 Å, whereas the corresponding screening length is about 100 Å. Moreover, one should point out that an increase in ionic strength reduces 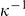 and increases the persistence length because the flexibility of the chain is also increased [29]. This simple picture can be easily extended to the case of finite concentration of DNA molecules by using the so-called PB cell model [35].

Aside from a cylindrical-like configuration to model a DNA chain, we also explore the case of the spherical shape in order to understand the effect of the configurational states on the denaturation of DNA. On the other hand, a mean-field approximation to the electrostatic effects in DNA molecules breaks down at short-distances between two double stranded DNA molecules due to the angular dependence of the electrostatic interaction as a result of helical charge distribution [17]. However, as we already discussed in the Introduction, a PB-cell description to obtain the osmotic pressure of DNA suspension, which is basically described by the atmosphere of the ions is shown to be in good agreement with experiments in the range of low to moderate DNA concentrations [16]. Within the cylindrical model, 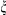 defines the type of DNA sequence.

Equation (14) is solved using the following boundary conditions,
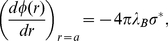
(15)

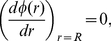
(16)where 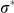 represents the charge density, i.e., the number of ions per area on the particle surface, which we here consider uniform. In the spherical case 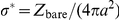 with 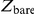 the number of ions on the sphere of radius 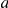 and 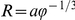 with 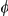 being the volume fraction, and in the cylindrical case 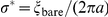 with 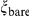 the linear charge distribution and 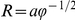. Equation (15) is simply Gauss’ law at the particle surface and equation (16) ensures electroneutrality inside the cell. From the numerical solution of the PB equation, one obtains the electrostatic potential at the cell edge, 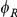, and can now proceed to compute the effective screening parameter: 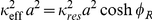. Additionally, the osmotic pressure from the pressure of the DNA molecules, 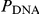, and the reservoir pressure, 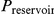, is 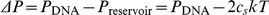 where 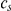 denotes the (monovalent) salt concentration in the reservoir. Applying this definition, the osmotic pressure of the suspension can be related to the effective screening parameter according to the relation [30],
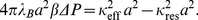
(17)


We should remark that the biomolecules in solution often respond to the effect of a pressure increase by reducing the partial molar volume of the system, hence the effect of pressure provides also useful information of hydration. It is related with the packing and distribution of cavities in the 3D structure of DNA inside the cell [25].

#### Osmotic pressure and compressibility

Since in this work we are particularly interested on the effects of the pressure variations on the denaturation processes that take place in a DNA suspension, it is convenient to introduce the so-called normalized osmotic isothermal compressibility [30],
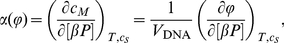
(18)where 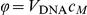 is the volume fraction of the DNA suspension and 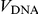 is the volume of a single DNA molecule. Hence, equation (18) can be straightforwardly evaluated once the osmotic pressure given by equation (17) is computed. Moreover, equation (18) is an important quantity because, on one hand, it represents the natural response of the system under changes of pressure and, on the other hand, it appears explicitly in our theoretical description as will see further below. In addition, two particular configurational DNA states, i.e., spherical and cylindrical, are considered.

Following the PB-cell model, the expression for the osmotic pressure is then described by equation (17). It depends on the salt concentration of the reservoir included in 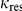 and the effective screening parameter, 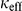, which is obtained by solving the PB-cell equation [35], [36]. We now present some representative results for both the osmotic pressure and the normalized isothermal compressibility for the different configurational states of the DNA molecule in order to illustrate, on one hand, the importance of the geometry and, on the other hand, the strong dependence of the thermodynamic properties on the medium characteristics. In this context, our results are not new, since the application of the PB-cell model in such a cases has been reported elsewhere, see, e.g., Refs. [30], [37]. Nonetheless, for the sake of the discussion, they are presented here to better understand the importance of the mechanical variations on the DNA denaturation, which will be discussed further below.

**Figure 3:**
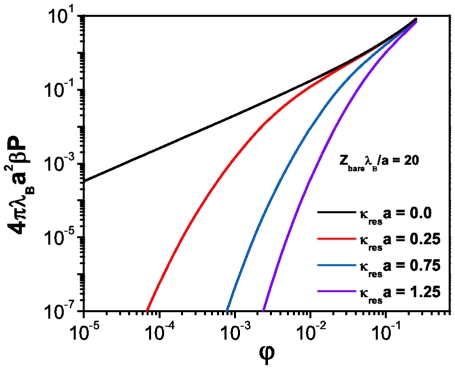
Osmotic pressure versus volume fraction for spherical macroions at different salt concentrations.

#### Spherical macroions

**Figure 4:**
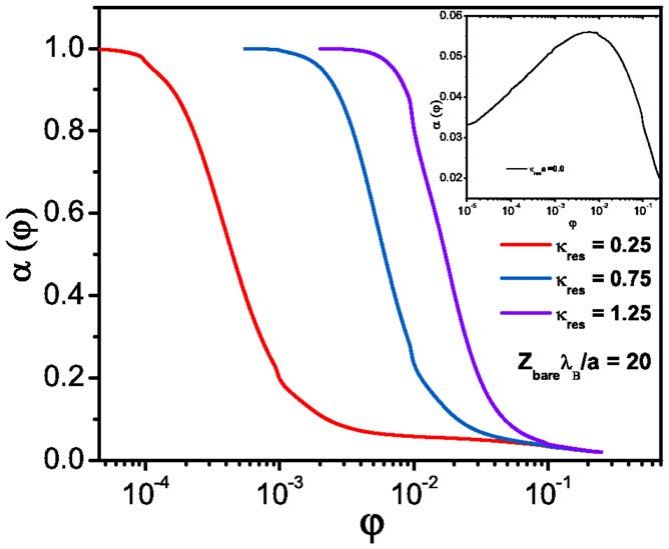
Normalized isothermal compressibility (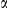) for spherical macroions at different salt concentrations. The inset corresponds to the no-salt case.

Volume fraction-dependence of the osmotic pressure is shown in Figure 3 for different salt concentrations in the case of the spherical state. We can see that the system’s pressure changes at low macroion concentrations, while by augmenting the biomolecules concentration, it takes nearly universal asymptotic values. This behavior can be understood as follows. In the low biomolecule concentration regime, electrostatic behavior becomes dominated by the screening effect of the salt, whilst in the high DNA concentration regime, 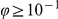, it turns the opposite way, i.e. the dominant thermodynamic effect is due to the excess of counterions [30]. As we already mentioned, one of the most important thermodynamic properties of charged suspensions is the normalized isothermal compressibility, 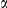. In Figure 4 we studied the effect of salt concentration and volume fraction in 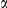 for the spherical case. We notice that in the 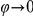 limit with salt, isothermal compressibility approaches its ideal gas value. On the other hand, the inset corresponds to the no-salt case 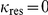. Here we observe a completely different scenario since 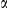 does not reach the ideal gas asymptotic regime, presenting instead a maximum close to 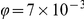. This effect is a natural cause of counterion dominance [30].

#### Cylindrical macroions

So far, we have discussed the EOS for a suspension made-up of spherical macroions. The case of charged cylindrical macroions is a direct extension of the spherical one. The infinite cylinder constant linear charge 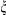 approximation is a very simple model that has been widely used to investigate the behavior of the EOS in this symmetry [16], [30], [37] that results relevant to our study since as a first approximation it can be used to model the DNA molecule [16].

**Figure 5:**
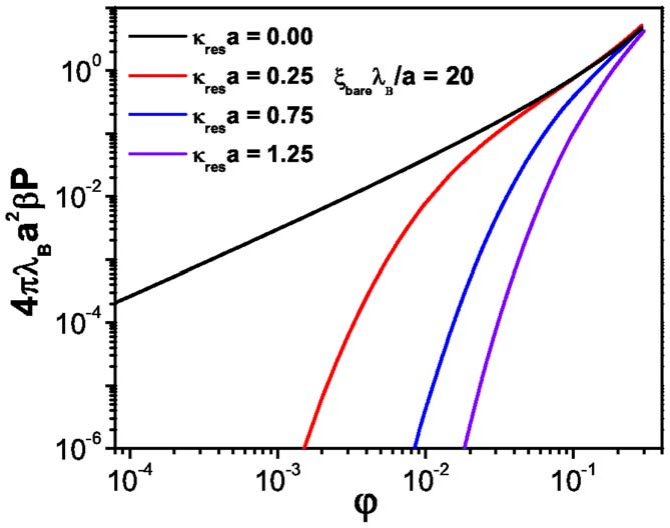
Osmotic pressure versus volume fraction for cylindrical macroions at different salt concentrations.

**Figure 6:**
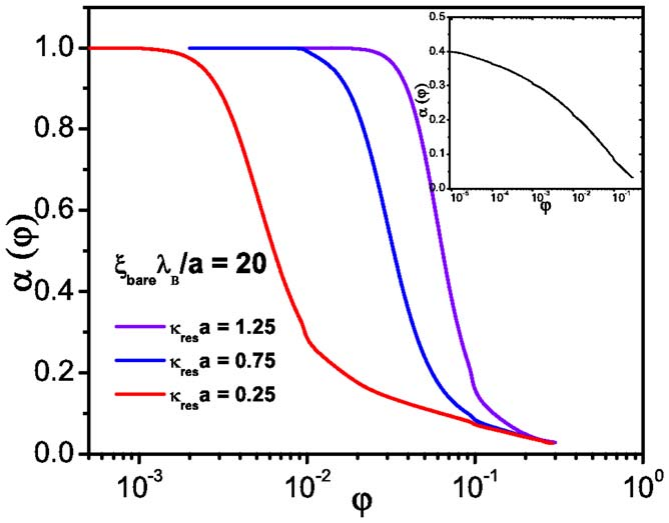
Normalized isothermal compressibility (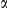) for cylindrical macroions at different salt concentrations. The inset corresponds to the no-salt case.

Figure 5 depicts the EOS for a cylindrical macroion suspension in the saturation regime, i.e. at charge densities 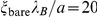, as a function of the volume fraction for different salt concentrations. We can see, as in the previous case, that salt dominates the osmotic pressure for low macroion concentration whereas this effect is reversed at high macroion concentrations where the contribution of the counterions is significantly superior. Our results are in excellent agreement with those available in the literature [37]. In Figure 6 we can observe the behavior of the normalized isothermal compressibility as a function of the volume fraction for different salt concentrations. In the added salt cases the compressibility behaves much as in the spherical case, with an ideal gas asymptotic regime in the limit 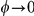. The inset also presents the no-salt-added case that unlike the spherical case does not show a well defined maximum but grows indefinitely. This mechanism is clearly due to a strong electrostatic coupling between the biomolecule and the counterions.

We have analyzed the role of the pressure in charged biomolecules in suspension and, in particular, we have seen the remarkable differences in those regions between the salt dominance and the counterions dominance. Hence, the microionic contribution, i.e., salt or counterions, to the osmotic pressure determines strongly the physical features of the system thermodynamics. This is a fundamental aspect since salt variations could lead to different scenarios of denaturation. Now, we will proceed to analyze the effect of the mechanical variations, i.e. compressibility, in the thermodynamics of DNA denaturation.

### Irreversible Coupling and Hysteresis in the DNA Melting Transition

One of the many interesting dynamic features of DNA melting is the presence of hysteresis. Interestingly, there are some instances in which DNA denaturation has assumed to proceed in a completely reversible way (e.g. [21]), but most commonly denaturation is described as showing different levels of irreversibility in the form of, for example, hysteresis curves [2], [3], [5], [6], [11]. It has been stressed [38] that, from a thermodynamical point of view, hysteresis shows up as the delayed response of the system’s structural features to a certain kind of energy-driven processes. This delay often is caused by dissipative couplings in an energy landscape enhanced by the presence of a multitude of metastable states. In the case of DNA, the very presence of an enormous number of spatial configurations, nearly equivalent in energetic cost, create a very complex topology of the energy landscapes, well beyond the usual double-well potential [2], [6], [38].

For example, with regards to *single stranded DNA* it has been mentioned that the presence of mechanic hysteresis is related to lower ionic concentrations whereas in higher salt concentration no hysteresis is observed [15]. This fact could be explained in terms of the relation between experimental observation times and internal relaxation times, as it was shown elsewhere [38]. Following the very same lines, it is possible to write down a linear response expression representative of the dissipative phenomena inducing hysteresis in the case of DNA denaturation [39].
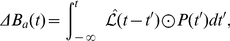
(19)where 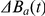 is the change of the fraction of broken hydrogen bonds during the melting process, 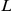 is a time propagation operator (a *memory kernel*), 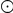 is an appropriated scalar product and 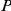 the stress distribution. The time propagation operator 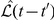 reflects the fact that cause (mechanical stress) and effect (denaturation, i.e. breaking of the hydrogen bonds between the double helix) cannot occur simultaneously. We can use a Green function kernel that up to first order takes the form [38]

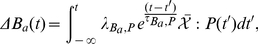
(20)with 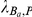 a cross-effect (Onsager-like) amplitude coefficient, 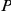 the stress tensor and 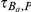 a relaxation time for the effect of stress (or pressure) on melting. 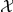 is a 2-tensor whose components are proportional to those of the propagation of stress. This tensor 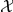 represents the *thermodynamic driving force* associated with momentum transfer. This expression is obtained by partial integration of a linear hyperbolic transport equation similar to the ones introduced by Maxwell, and later by Cattaneo and Vernotte (MCV) [40], [41]. This is important because the (extended) irreversible thermodynamics formalism is consistent with such transport equations [26], [27], [38].

The output (change in normalized hydrogen bond composition) is related with the actual input (change in the stress distribution) via a dynamic propagation operator of *irreversible* nature. This operator is then associated with dissipation and is characterized by a relaxation time. This irreversible phenomenon (of causality) is also present in the case of the coupling between various physical processes due to the presence of a conducting medium that creates a delay between the onset of a field and its response (in the form of the correspondent *coupled effect*). The experimental observation of hysteresis depends on the ratio of this relaxation time with the relaxation time associated with the kinetic mechanism of DNA melting.

Let us consider the linear expression for the change in normalized concentration due to stress (equation (20)). We define the *stochastic time derivative* [42], [43] of this equation with a proper time 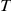 as
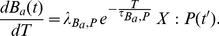
(21)


In the thermodynamic limit 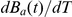 equals 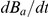, and this in turns gives a relation for the relaxation time 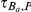 in terms of the proper time 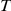. If we identify this stochastic proper time 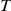 with the observation time, we note that the ratio of relaxation time to observation time 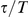 depends on system specific properties such as magnitude of the stress, chemical potential and uncompensated heat production, all of these weighted by the amplitude 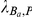. Because of this, for some systems (i.e., lower salt concentration or as we will show later *cylindrical* DNA structures) it is possible to experimentally observe hysteresis while for other processes (higher salt concentration or *quasi-spherical* DNA configurations) it is not. If the ratio of relaxation to observation times is closer to unity then hysteresis shows up as a manifestation of this *dynamical coupling*.

Of course, in a living cell under physiological conditions the main mechanism of mechanical stress redistribution (momentum flow) is by means of hydrostatic pressure variations. As we discussed previously, the osmotic contribution to the pressure, coming from the electrostatic interactions, could be treated by using the PB-cell model. In order to introduce the PB-cell equation of state into the thermodynamic formalism, let us recall the rate of energy dissipation (equation (3)) that in the case of the coupling between the concentration (fraction) of broken hydrogen bonds and the hydrostatic pressure reads as follows:
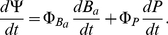
(22)


This equation describes the rate of hydrogen bonds disruption as a function of the change in pressure via an irreversible coupling given by a constant (steady state) non-equilibrium entropy production. Time integration of equation (22) (defining 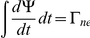) gives us
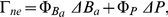
(23)but 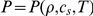 and also 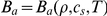, i.e., they are state functions of the medium, so that,
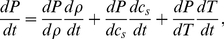
(24)

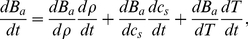
(25)


From equation (22) one is able to see that 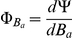 plays the role of a *chemical potential* and that 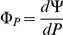 is the negative of the molar volume. From equation (25) one immediately obtains,
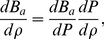
(26)

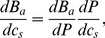
(27)

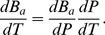
(28)


The calculation of 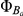, the chemical potential associated with hydrogen bond disruption, could proceed as follows. Recalling that 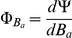 a Maxwell relation states that 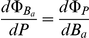 so that one can write down,
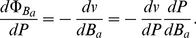
(29)


Then, one is able to calculate 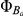 as follows,
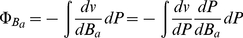
(30)which gives the relation,
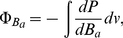
(31)but 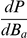 is just 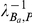 so that,
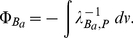
(32)


If we introduce the fact that 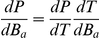 and that the dependence of the rate of broken hydrogen bonds by temperature 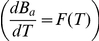 is known both experimentally and from models as the one proposed by Poland and Scheraga [44], [45], one finds an expression for 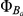 in terms of the system equation of state as,
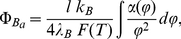
(33)with 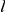 the length of the DNA sequence and 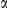 the normalized isothermal compressibility. The negative molar volume, 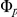, for the cylindrical geometry could be expressed as,
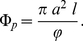
(34)


Inserting equations (33) and (34) into equation (23) leads to the relation,
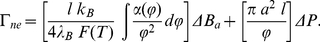
(35)


It is just equation (35) which gives us the opportunity to consider the coupling between 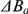, the change in the fraction of broken hydrogen bonds, and 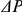, the pressure change as the origin of the observed hysteretic behavior. Moreover, we should recall that the balance (steady state) described in equation (35) is only possible due to the competition between both terms of the r.h.s. In particular, the factor of the first term clearly demonstrates that such balance depends on the ratio between the variation of the pressure at constant temperature, i.e., the compressibility, and the fraction of broken hydrogen bonds due to thermal variations. It is therefore interesting that our approach accounts for DNA denaturation using well-controlled parameters, such as pressure.

We should remark that equation (35) is thus the cornerstone of our theoretical formulation. It is a one parameter (energy-dissipation) model based on an irreversible thermodynamics formalism and an equilibrium mean-field approximation. Both schemes allow us to study denaturation of DNA biomolecules. This kind of coupling leads to a particular way of combining different formulations to give an alternative description of a very complex phenomenon as the denaturation of biopolymers. To obtain equation (35) we have assumed several approximations; one of them considers a specific shape and arrangement of DNA molecules. This specific condition can be relaxed and, strictly speaking, one could use a more realistic description, but, from computational point of view, highly demanding, model for DNA molecules in suspension. Therefore, by using a PB-cell approximation we are able to capture the main features of the electrostatic interactions between charged molecules that lead to the breaking of hydrogen bonds during denaturation.

## Results and Discussion

We have introduced the formalism and tools needed for a systematic study of the denaturation process in biomolecules with either spherical or cylindrical shape. In particular, we have derived a relation for the fraction of broken hydrogen bonds due to mechanical variations. We also calculated the related EOS for these systems within the PB-cell approximation and showed how the reduced EOS is given by the osmotic pressure itself, as it can be seen in equation (17). With these necessary input parameters we can proceed to carry out a mean-field analysis of DNA denaturation processes.

To make explicit calculations and at the same time maintaining the discussion as *general* as possible, we decided to use a random DNA sequence of size 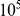 base-pairs (a somehow *typical* gene scale) for the calculation of the composition-dependent thermal denaturation function 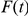 [45]. We did so, in order to leave-out (at the moment) biophysical particularities that different DNA sequences possess. Nevertheless, we have started a systematic research project to characterize mechanical denaturation phenomena for a variety of genomic sequences in a systematic way. Also in order to built universal denaturation curves, we have decided to use a dimension-less version of equation (35). If we define 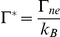 it is possible to do so.

To monitor the melting of a suspension of charged spherical or cylindrical biomolecules we start from equation (35). In order to trace the denaturation process it appears more convenient to rewrite this expression in terms of the osmotic pressure to obtain:
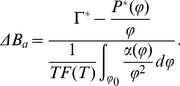
(36)


From equation(36) we can see that the denaturation process arises by a dissipative competition between entropy production and osmotic pressure effects. In this sense, osmotic pressure *dynamically* stabilizes DNA via a non-equilibrium coupling. If we look at equation (35), the first term at the r.h.s is related to dissipative thermal processes related to denaturation (as given by the 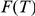 contribution) whereas the second term at the r.h.s. is related to purely mechanical (i.e. osmotic) effects. If we consider the physical limits of equation (36), we can notice that 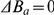, the system is in a non-denaturated state whereas 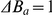, implies a fully denaturated state with the two strands completely separated. The scenario 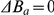 corresponds to perfect balance between thermal dissipation and osmotic effects. 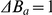 implies a (relative) absence of osmotic contributions, thus allowing *free* thermal denaturation. It is known that hysteresis arises because of the competing dynamics of several dissipative processes [38], [39]. In this case the driving force is the competition between thermal dissipation and mechanical effects of osmotic origins.

**Figure 7:**
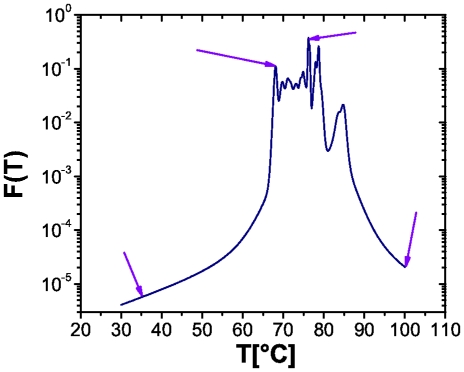
Plot of the fraction of broken hydrogen bonds as a function of temperature F(T) for a random DNA sequence. Arrows indicate temperature values considered in this study. Plot obtained by using MeltSim 1.0 [45].

In our study we limited ourselves to a pure random nucleotide sequence in order not to introduce any chemical composition bias. A sequence-dependent study will be left for a future systematic study to investigate the denaturation in systems with a particular biological scope in mind. However, the actual calculations in those cases are straightforward. The sequence reported here was the same used to calibrate the program MELTSIM [45]. In Figure 7 we show the temperature dependence of the fraction of broken hydrogen bonds in this sequence. Arrows indicate the temperature values considered in this work.

### Denaturation of Spherical Biomolecules

**Figure 8:**
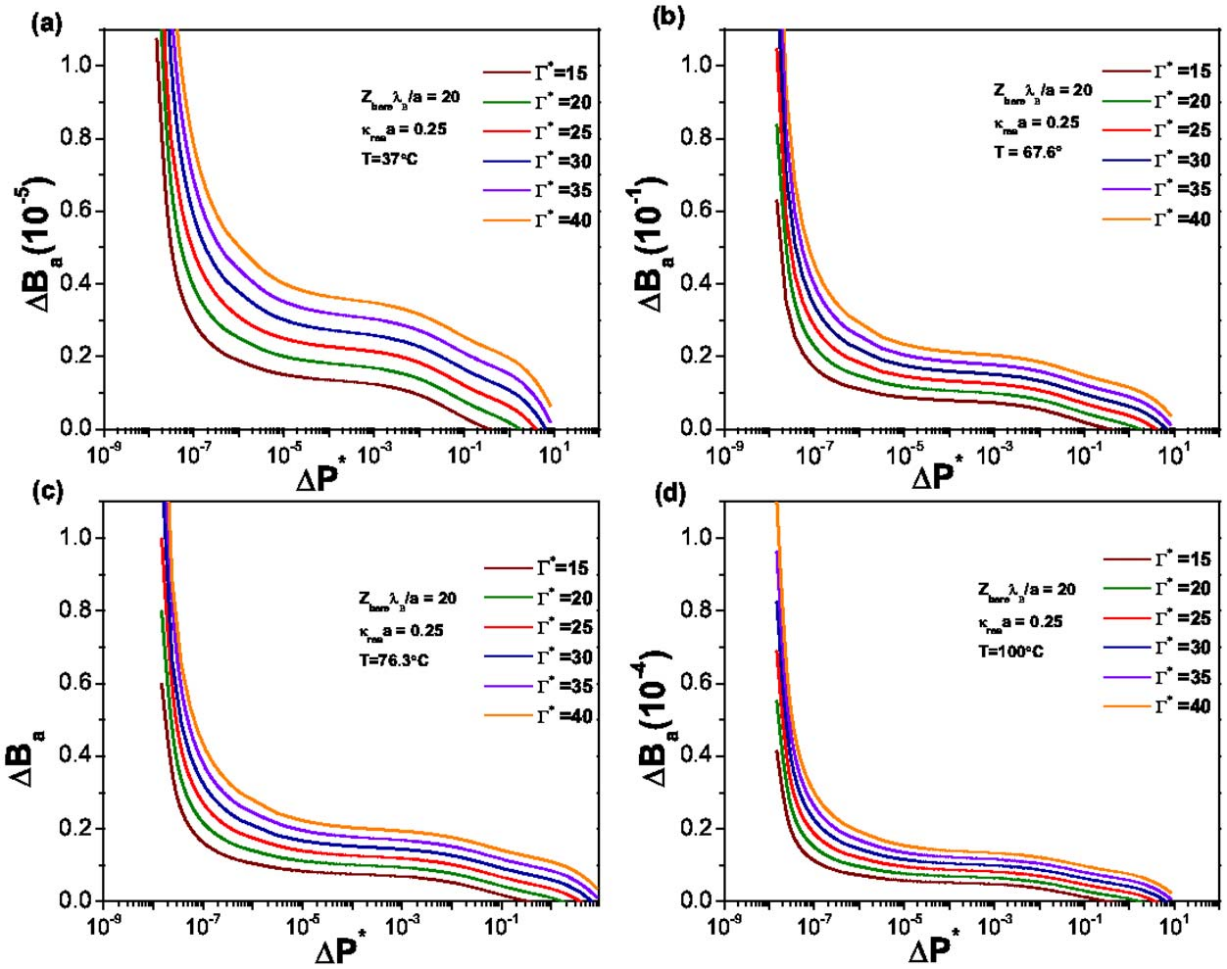
Denaturation curves for spherical biomolecules in suspension. The value of 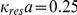, Temperature is as follows: (a) 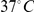, (b) 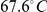, (c) 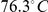 y (d) 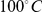. All plots correspond to the same sequence.

In Figure 8 we can see the denaturation curves for spherical biomolecules in a suspension with 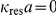.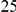 and in the saturation regime, i.e., 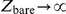. We plot the fraction of broken hydrogen bonds (unpaired bases) as a function of the osmotic pressure considering the coupled effect with the temperature as already mentioned above. Equation (36) depends on 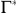, which represents the reduced form of the energy dissipation in the process, so we plot several curves corresponding to different values of the dissipation 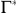. 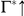 implies that a lot of energy is being dissipated by the system in a fast process, hence no hysteresis effect is expected. 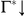 points to a very slow process with small dissipation so no hysteresis is shown either. Hence, 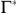 is a measure of the possibility of hysteretic behavior in the system. With regards to the experimental measurement of 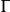 (or 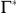) it is likely that techniques such as microcalorimetry at a single cell level, and especially Isothermal Titration Calorimetry (ITC) could be applied on a real-time basis to monitor changes in local thermodynamics within the cell [46]. These experiments will also shed light in the thermal component of biomolecular dynamics, and thus will serve to fine-tune the predictions of the nonequilibrium thermodynamics model presented here.

**Figure 9:**
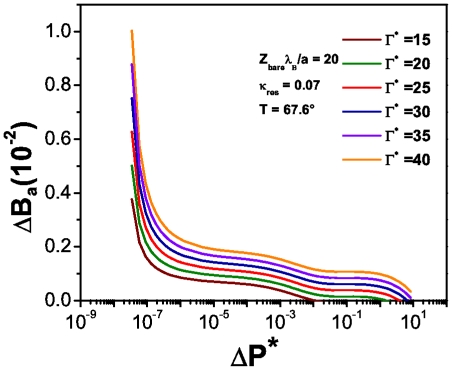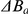, the fraction of broken hydrogen bonds as a function of the reduced osmotic pressure for spherical biomolecules. This plot corresponds to the low-salt regime 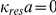.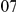 and under charge saturation conditions and at T = 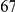.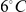 (i.e. the melting temperature for this sequence).

In order to show the effect of the ionic strength in the denaturation process we present melting curves for a low salt concentration (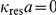.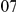) (Figure 9). If we compare Figure 8b with Figure 9 we can observe that in higher salt concentration, Figure 8b, the number of broken hydrogen bonds is much larger than those in a system with a lower salt concentration. According with equation (36) we can see that the difference between both scenarios is given precisely by an osmotic pressure contribution since this affects the dissipation rate.

**Figure 10:**
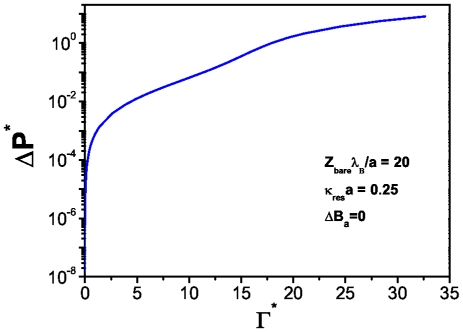
Reduced osmotic pressure versus energy dissipation for(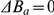) [No hydrogen bond broken] in spherical biomolecules.

In Figure 10 we show the dependence of the osmotic pressure with the energy dissipation in a non-denaturated state. This is a very interesting case since it can be observed a *threshold osmotic pressure* at which the system starts to dissipate energy. A non-trivial behavior is observed as given by a non-monotonic growth of the osmotic pressure as a function of the dissipation rate. At low energy dissipation the osmotic pressure exerted needs to break an energy barrier to preserve the hydrogen bonds unbroken. Four different regimes are apparent: first a really steep growth followed by a curved region, then a linear increase and finally what it looks like a forming plateau. These different regimes are a consequence of the nonlinear behavior of the solutions of the PB differential equation, which predicts a non-monotonic dependence in the pressure, as already shown in Figure 3.

### Denaturation of Cylindrical Biomolecules: DNA

We now discuss the results for a cylindrical model intended to represent, although in a highly simplified manner, double-stranded helical DNA (ds-DNA). Following a procedure akin to the one we used in the spherical case, the same set of parameters was used in order to highlight the differences due to geometrical effects. A noticeable difference, however, is that in this case we have considered a linear charge density instead of the total charge. This will be specially useful when performing sequence-specific calculations.

**Figure 11:**
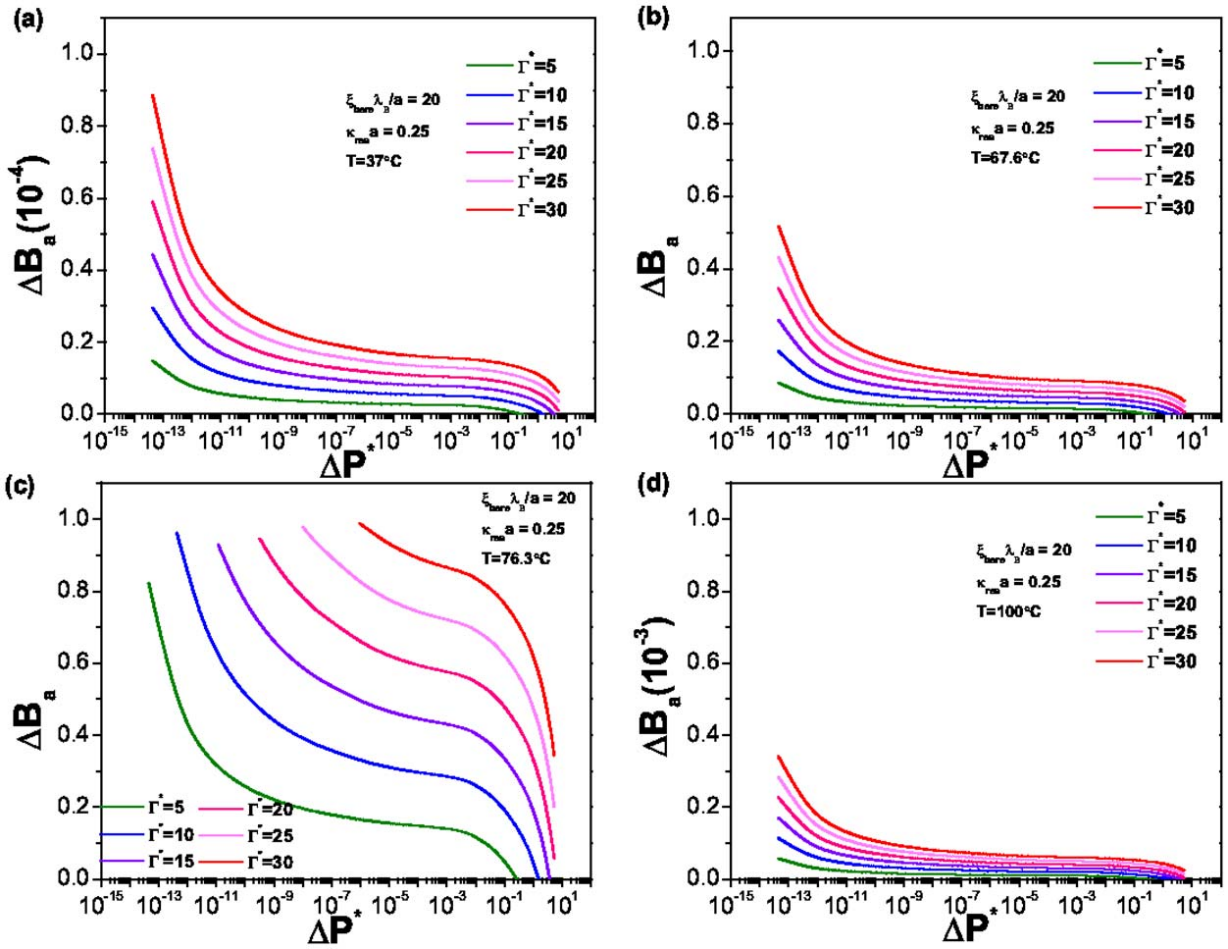
Denaturation curves for spherical biomolecules in suspension. The value of 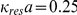, Temperature is as follows: (a) 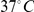, (b) 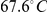, (c) 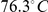 y (d) 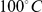. All plots correspond to the same sequence.

In Figure 11 we show the denaturation curves for the case of cylindrical biomolecules in a suspension characterized by a value 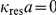.25. Just as in the spherical case, the curves show a strong temperature dependence which is related with the number of broken hydrogen bonds within the molecule. Unlike the spherical molecules, however, not all the curves follow the same behavior, a fact specially clear in Figure 11. It presents different curvatures, also, the system is able to attain fully denaturated state. These two issues point to a probable signature of hysteretic behavior, in particular, for relatively high values of energy dissipation, namely, 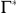 = 5 and 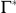 = 10.

**Figure 12:**
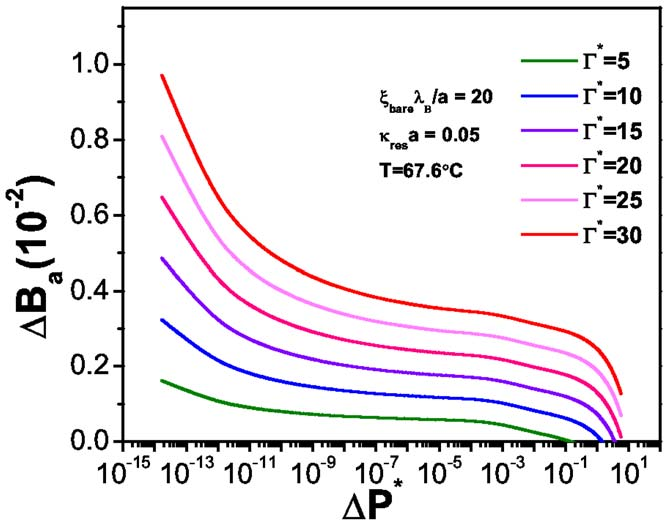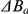, the fraction of broken hydrogen bonds as a function of the reduced osmotic pressure for cylindrical biomolecules. This plot corresponds to the low-salt regime 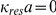.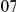 and under charge saturation conditions and at T = 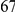.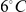 (i.e. the melting temperature for this sequence).

**Figure 13:**
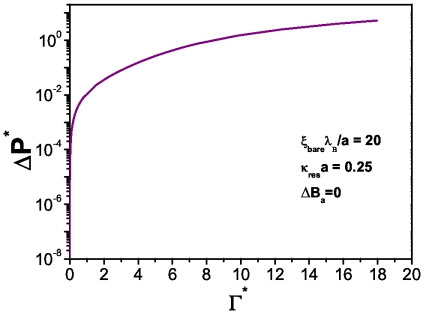
Reduced osmotic pressure versus energy dissipation for(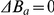) [No hydrogen bond broken] in cylindrical biomolecules.

Figure 12 shows the corresponding denaturation curves, under a lower salt concentration (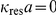.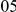) at 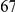.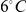. If we compare this plot with Figure 11b we will see the effect that salt concentration has in the number of unpaired bases in the biomolecule: the higher the concentration of salt in the system, the bigger the number of broken hydrogen bonds at a given temperature and energy dissipation rate. This fact relates denaturation with an osmotic pressure effect. In this regard, Figure 13 presents the behavior of the osmotic pressure as a function of energy dissipation at zero denaturation. It shows a threshold value for the osmotic pressure of about 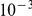 at which the system starts to dissipate energy. Unlike the spherical case, this threshold value is larger and energy dissipation lower due to the particular structure and charge density of the biomolecule.

**Figure 14:**
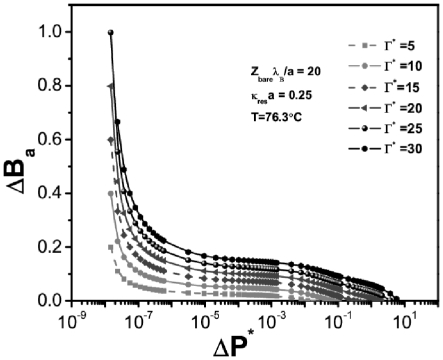
Denaturation curves for spherical DNA cells. Typical parameters of charge for globular biomolecules, 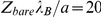, salt concentration, 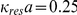 and temperature, 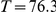
°C, are considered.

The difference between the behavior of the quasi-spherical biomolecules and the cylindrical ones lies precisely in their different response to dissipation. In Figure 14 we show melting curves for entangled DNA molecules forming globular structures (to approximate the compact chromatin structure of DNA in its inactive state) for several values of dissipation 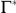, for denaturating conditions of salt, and temperature. It is possible to notice in the different curves in Figure 3 that the effect on hydrogen bond disruption is already very small (as it would be physically and biologically expected, of course), as is also small the effect of increasing the energy dissipation rate 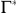. For this reason, even if the model allows for hydrogen bond breaking and energy dissipation, no noticeable hysteretic effects could be found. We are able to see that under the model for the equation of state considered here (PB-cell), tightly-entangled DNA is able to melt and even under moderately high values of dissipation the transition shows no hysteresis. Different denaturation paths are almost absolutely parallel and very close to each other in such a way that one can consider the phase transition as essentially reversible. This is of course an expected result from the biological stand point, in this highly compact arrangement within the chromatin structure, DNA would in fact melt and re-hybridize with no apparent change, hence preserving its valuable information content.

**Figure 15:**
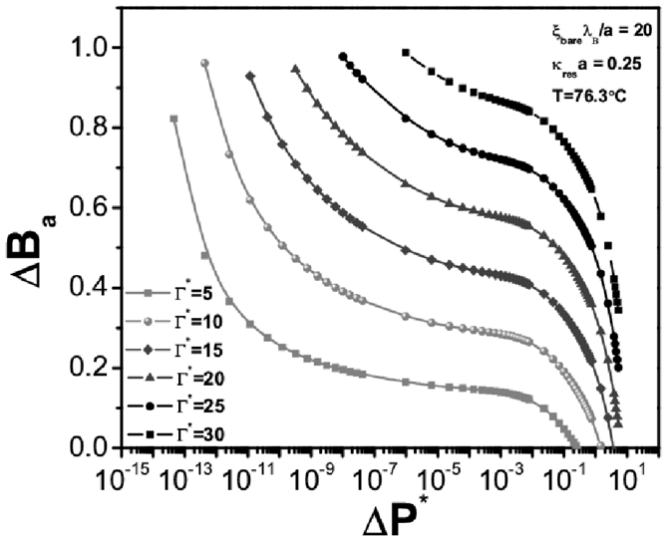
Denaturation curves for cylindrical DNA cells. Typical parameters of linear charge density, 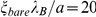, salt concentration, 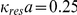 and temperature, 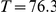
°C, are considered.

In stark contrast, once DNA has the random coil configuration (here simplified as a very long cylindrical robe) needed for its replication and transcriptional duties; it is more exposed to the influence of different electro-hydrodynamical interactions within the subcellular medium. In this scenario, we can consider the melting curves given in Figure 15. For the same denaturating conditions, as the previous case (depicted in Figure 14), it is possible to observe that, under the very same dissipation rates 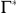, the behavior is quite distinct. In the first place hydrogen-bond breaking occurs to a much higher extent than in the globular (sphere-shaped) case, as we have stated this is so because the molecule is more exposed to denaturating interactions. But, what is more important for our present argument, is that the curves are not parallel to each other. The plots show also noticeable distances so that different denaturation paths (tagged by different energy dissipation rates 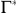) would lead to hysteretic behavior.

Within the possible mechanical explanations for hysteresis in DNA stretching denaturation, one of the most likely is that of unpeeling of one strand from the other, beginning from nicks in the phosphate backbone; this has been suggested in the past [6], [15], [47]–[49]. Hysteresis under these conditions represent an interesting phenomenon, since DNA molecules in their active states within the cell are constantly denaturating and re-naturating (sometimes even due to local thermal and electro-hydrodynamical fluctuations). The fact that they undergo hysteresis could lead to an actual structural difference (maybe a *hairpin* or any other kind of mutation) that could, in some instances affect its biological function. As we have mentioned earlier, there is a potential relation with the phenomenon of stress-induced DNA damage that has been related with cellular aging [50] and disease effects [51], [52].

To summarize, in this work we have presented the main elements of our theoretical model to describe the phenomenon of DNA denaturation and illustrated its applicability with model DNA molecules. However, the model can be used to systematically investigate real sequences under physiological conditions. Furthermore, our approximation is able to incorporate naturally more accurate equations of state that take into account properly the complex (helicoidal) structure of the DNA [17] or correlation effects between microions when the suspension is in contact with asymmetric salt reservoir.

### Conclusions

The process of DNA denaturation or melting has been studied extensively in the last decades. However most of these studies lie at the single molecule level. Now it is becoming clear that this process depends on many-body interactions of a highly complex nature whose ultimate behavior can only be traced-back by means of a molecular thermodynamics approach. This is so in order to establish a level of description that will include both molecular and macroscopic components of the phenomenon. Here we present a theoretical framework that allows us to establish what is the relation between molecular interactions of electrostatic origin and physicochemical properties of biomolecules. In particular, we focused in the denaturation of DNA in suspension due to variations in the osmotic pressure. By means of analyzing the dissipative processes involved, it has been possible to elucidate some key elements of the structural stability of this biomolecule which are related with the presence of hysteresis. These effects are of an irreversible nature and could be connected with important biological processes such as the ones behind DNA damage. The origins of DNA damage are extremely important from the biomedical standpoint.

We have proposed a theoretical framework to describe DNA melting process by a combined approach using an irreversible thermodynamics formalism and a mean-field Poisson-Boltzmann theory under a so-called cell approximation. Such approximation is valid in the weak electrostatic coupling regime, i.e. for systems with a moderate charge. By means of this framework, we were able to study the phenomenon of hysteresis in DNA melting -under the given assumptions- in a systematic way. The results show that DNA melting was strongly dependent in temperature and salt concentration in the suspension -as it was already known for decades-, but also in the associated energy dissipation rate, pointing out to a balance between thermal, mechanic and electrostatic forces. Even if the results presented here correspond to an arbitrary sequence, the proposed methodology is useful to analyze systems of biological/biomedical interest. This study opens up a new way of combining statistical mechanics of soft condensed matter and non-equilibrium thermodynamics. This avenue of research could have an impact in the study of complex structured fluids, in particular solutions and suspensions of biomolecules. By incorporating studies of (somehow realistic) equations of state for these biological systems in a solid theoretical framework given by irreversible thermodynamics, it may become possible to establish the foundations for modeling biophysical phenomena under a general Systems Biology-like scheme. Natural perspectives of further development for this work would be: development of more realistic models for the equations of state, and also the incorporation of fine sequencing (and re-sequencing) data for biologically relevant genomic segments. Work along these lines is currently in progress.
